# Hematological Malignancy Specific Patient-Reported Outcome Measure (HM-PRO): Construct Validity Study

**DOI:** 10.3389/fphar.2020.01308

**Published:** 2020-09-08

**Authors:** Pushpendra Goswami, Esther N. Oliva, Tatyana Ionova, Roger Else, Jonathan Kell, Adele K. Fielding, Daniel M. Jennings, Marina Karakantza, Saad Al-Ismail, Graham P. Collins, Stewart McConnell, Catherine Langton, Magda J. Al-Obaidi, Metod Oblak, Sam Salek

**Affiliations:** ^1^ School of Life and Medical Sciences, University of Hertfordshire, Hatfield, United Kingdom; ^2^ Haematology Unit, Grande Ospedale Metropolitano, Reggio Calabria, Italy; ^3^ St. Petersburg State University Medical Center and Multinational Centre for Quality of Life Research, St. Petersburg, Russia; ^4^ Patient Research Partner, Milton Keynes, United Kingdom; ^5^ Cardiff and Vale University Health Board, Cardiff, United Kingdom; ^6^ University College London Cancer Institute, London, United Kingdom; ^7^ Royal Surrey County Hospital NHS Foundation Trust, Guildford, United Kingdom; ^8^ Leeds Teaching Hospitals NHS Trust, Leeds, United Kingdom; ^9^ Singleton Hospital, ABM University Health Board, Swansea, United Kingdom; ^10^ Oxford University Hospitals NHS Trust, Oxford, United Kingdom; ^11^ West Middlesex University Hospital, Isleworth, United Kingdom

**Keywords:** hematological malignancy, HM-PRO, quality of life, symptoms, construct validity, clinical practice, clinical research

## Abstract

**Background:**

Validity is the ability of an instrument to measure what it claims to measure. It means the degree to which the empirical evidence supports the trustworthiness of interpretations based on the calculated scores. The hematological malignancy (HM) specific patient reported outcome measure (HM-PRO), is a newly developed instrument for use in daily clinical practice as well as in research. This study, provides the evidence for construct validity of the HM-PRO, specifically focusing on the convergent and divergent validity compared to the other established instruments used in hematology.

**Methods:**

This validation study adopted a prospective cross-sectional design where a heterogeneous group of patients diagnosed with different HMs and different disease state were recruited. A total of 905 patients were recruited from seven secondary care hospitals in the UK and online through five patient organizations. Patients were asked to complete the HM-PRO and other cancer specific PRO’s, FACT-G and EORTC QLQ C-30. Data analysis was performed using IBM SPSS 23 statistical software.

**Results:**

A total of 486 males (53.7%) and 419 females (46.3%), with a mean age of 64.3 (± 12.4) years and mean time since diagnosis of 4.6 ( ± 5.2) were recruited. The total score of Part A of the HM-PRO highly correlated with the five functional scales of the EORTC QLQ-C30 (Physical = −0.71, Role = −0.72, Emotional = −0.64, Cognitive = −0.58, Social = −0.74—p < 0.001). With respect to correlation with FACT-G, the total score of Part A of the HM-PRO highly correlated with Physical (−0.74), Emotional (−0.57), Functional (−0.66) domains and overall score of FACT-G (−0.74). Similarly, the total score of Part B of the HM-PRO highly correlated with three symptoms scales of EORTC QLQ-C30 (Fatigue scale = −0.74, Nausea and Vomiting = −0.52, Pain = −0.59—p < 0.001) and individual symptom items (Dyspnea = 0.51, Insomnia= 0.43, Appetite loss = 0.54—p < 0.001).

**Conclusion:**

The construct validity evidence presented in this research is a testimony to the HM-PRO’s ability to measure HRQoL issues which it intends to measure. This is of utmost importance when a PRO is used in routine clinical practice so that the interpretation of the scores or response to an individual item is understood by the clinicians/nurses as intended by the patients.

## Introduction

Hematological malignancies are a cause of morbidity and mortality. Although, treatments have the potential to cure or prolong life, both the disease and the treatment may cause substantial suffering. The main goal has been “cure” for many years, however, more recently, equal emphasis has been placed on patients’ “quality of life.” Measuring quality of life is not only a new clinical end-point for cancer treatments (Kosmidis, 1996; Allart-Vorelli et al., 2015), but is also used to guide the decision-making in daily clinical practice (Esser et al., 2018). The rapid growth in terms of the availability of the new treatments for hematological malignancies has added more complexity in the decision-making process. The use of patient-reported outcomes (PROs) both in clinical research and routine practice can help to collect this complex information from patients in a systematic manner to be used as an aid to treatment decision-making process (Efficace et al., 2017; Esser et al., 2018). With such an importance attached to PROs both in clinical research and routine practice, special attention should be paid to the development and validation of such instruments.

Validity, reliability, and responsiveness are the three measurement properties which should be assessed according to the COSMIN study conducted by Mokkink et al. (2010) to reach international consensus, to prove that a newly developed health-related quality of life instrument (HRQoL) possesses strong psychometric measurement properties (Mokkink et al., 2010). Validity is the ability of an instrument to measure what it claims to measure (Wan, 2002; Roberts and Priest, 2006; Mokkink et al., 2010; Fayers and Machin, 2013). It means the degree to which the empirical evidence supports the trustworthiness of interpretations based on the calculated scores (Messick, 1994). To provide evidence for validity is one of the essential steps in the development of an instrument for quantifying HRQoL and to prove the legitimacy of the instrument. The validity attribute relates to particular use of the scale and is not an inherent trait of the instrument (Messick, 1988; Zamanzadeh et al., 2015). There are different ways to assess construct validity including demonstration of moderate to high correlation with a standardized instrument measuring the same concept to show “convergent validity,” or low correlation with an instrument measuring different concept to show “divergent validity.”

The hematological malignancy specific patient reported outcome measure (HM-PRO), is a newly developed instrument for use in daily clinical practice as well as in research (Goswami et al., 2016; Goswami et al., 2018a; Goswami et al., 2018b; Goswami et al., 2019a; Goswami et al., 2019b; Goswami et al., 2020). The HM-PRO is undergoing all necessary assessments to meet the minimum standards set out by regulatory authorities such as the FDA. This study, provides the evidence for construct validity of the HM-PRO, specifically focusing on the convergent and divergent validity compared to the other established instruments used in hematology.

## Methods

### Ethics

Multicenter ethics approval was obtained from the National Research Ethics Service (NRES) South West Bristol, UK (ref 14/SW/0033) followed by individual “research and development” approvals from all the participating centers. A signed informed consent was obtained from all the study participants.

### Study Design

This validation study adopted a prospective cross-sectional design to which a heterogeneous group of patients diagnosed with different HMs and different disease state were recruited. In the absence of a gold standard, the study design for carrying-out the HM-PRO validity, hypothesis testing approach was adopted. The patients were recruited from inpatient and outpatient clinics of seven secondary care hospitals in the United Kingdom and gave informed written consent in person or online if they were recruited through hematology/oncology patient organizations (Myeloma UK, Leukemia Care, Lymphoma Association, MDS support group, and MPN foundation). The patient organization posted the summary of the project on their news page, trial page, and other social media. The summary was provided with the link to the patient information sheet and contact details of the research team. Those who were interested gave online consent and provided their demographic information together with contact details. All patients were then sent a set of three questionnaires with a free post envelope to return the completed instruments. The inclusion criteria for the participants were: adult patients diagnosed with any HM as per latest WHO classification; at any stage of the disease; at any stage of the treatment; and able to read and understand English. The exclusion criteria were: unable to read and understand English and unable to give written informed consent.

### Instruments

#### HM-PRO

The hematological malignancy–patient reported outcome measure (HM-PRO) is a newly developed composite measure consisting of two scales: Part A (Impact); and Part B (Signs and Symptoms). Part A measures the impact of the HM and its treatment on a patient’s HRQoL, and Part B captures the severity of disease or treatment related signs and symptoms (Goswami et al., 2016; Goswami et al., 2017; Goswami et al., 2020). Part A has a total of 24 items in four domains: physical behavior (7); social behavior (3); emotional behavior (11); and eating and drinking habits (3). Patients’ responses are recorded on a three-point Likert scale (Not at all, A little, A lot) and “not applicable” as a separate response option. Part B consists of 18 items in a single domain and the responses are captured on a three-point severity Likert scale (Not at all, Mild, Severe). The third item of the “Eating and Drinking habits” domain in Part A i.e. “My drinking habits have changed,” and ninth item of Part B related to “skin problems” are not included in the scoring system but collected for additional information. The HM-PRO has shown good reliability with Cronbach’s alpha and ICC with coefficient greater than 0.8 for all four domains of Part A and for Part B (Goswami et al., 2016; Goswami et al., 2017; Goswami et al., 2020).

#### EORTC QLQ C-30

European Organization for Research and Treatment of Cancer Quality of Life Questionnaire-Core 30 (EORTC QLQ-C30) is one of the most widely used cancer specific HRQoL instruments including for patients with HM (Kvam et al., 2010; Kvam et al., 2011). This instrument has a total of 30 items in five multiple item functional scales: Physical (5), role (2); emotional (4), cognitive (2), and social (2); two global health and quality of life (QoL) items, three symptom scales (Fatigue—3, Nausea and Vomiting—2, and Pain—2); and six single items (Dyspnea, insomnia, appetite loss, constipation, diarrhea, and financial difficulties). Responses are recorded on a four-point Likert scale (Not at all–Very much). All the scales and single items are measured on linear scores ranging from 0 to 100 (Aaronson et al., 1993; Knobel et al., 2003).

#### FACT-G

Functional Assessment of Cancer Therapy- General (FACT-G) is another widely used cancer specific HRQoL including for patients with HM (Yost et al., 2013). This instrument has a total of 27 items in four domains: physical well-being (7); social/family well-being (7); emotional well-being (6); and functional well-being (7). Responses for all the items are recorded using a five-point Likert scale (Not at all–Very much). The total score for all the items is calculated on a linear scale ranging from 0 to 100 (McQuellon et al., 1997; Cella et al., 2012; Hlubocky et al., 2013; Yost et al., 2013).

#### Global Question

Global Question (GQ) is a general question which assesses the overall impact on HRQoL from a patient’s perspective. The response to the question is captured using a five-point Likert scale (extremely large effect on my life to no effect on my life).

### Data Processing and Analysis

The data collected from the in-patient and out-patient clinics of the seven secondary care hospitals in the UK were manually entered and 20% of the entered data were randomly selected and cross validated. For the remaining data collected through online platform, it was possible to have the direct data entry to the database minimizing the potential human error (Dillman, 2006). Data analyses were carried out with IBM SPSS 2, statistical software. The following tests were performed on the data collected: descriptive statistics to explore distribution of the variables and the HM-PRO scores; Spearman’s Rank correlation coefficient was calculated to establish the relationships between the scores of both Part A and Part B of the HM-PRO and other measures. According to Fayers and Machin (2007), a correlation coefficient of greater than 0.3–0.4 supports convergent validity (Fayers and Machin, 2007). Furthermore, univariate Ordinary Least Squares (OLS) regression analysis was performed to assess the relationship between scores of the HM-PRO and the other two instruments. To assess how much of the variance in the independent variable is explained by the predictor variable, R^2^, the coefficient of determination was used (Streiner et al., 2015); and to determine the predictors of the HRQoL in hematological malignant patients, multivariate OLS regression was performed. The score of HM-PRO was the dependent variable, and patient demographics, diagnosis, and disease state were the independent variables.

## Results

### Patient Demographics

A total of 905 patients were recruited from seven secondary care hospitals in the UK. This included 486 males (53.7%) and 419 females (46.3%), with a mean age of 64.3 ( ± 12.4) years and mean time since diagnosis of 4.6 ( ± 5.2) (**Table 1**). The diagnoses were: acute leukemias—lymphoblastic (n = 29) and myeloid (n = 67); chronic leukemias—lymphoid (n = 64) and myeloid (n = 45); multiple myeloma (n = 296); Non Hodgkin Lymphomas—indolent (n = 41) and aggressive (n = 54); Hodgkin Lymphoma (n = 37); myelodysplastic syndromes (n = 158); and myeloproliferative neoplasms (n = 114) (**Table 1**). The highest number of patients with MM (10) and ANHL (10) were in the age group of 60–70 years. Fifty-nine percent of patients did not report any comorbidities. With respect to the employment status, the highest percentage of patients (61.7%) were retired, followed by 27.88% who were employed and working fulltime (**Table 1**), and ten were students.

**Table 1:** Sociodemographic characteristics of the study participants.

n = 905			
**Age (Years)**	Median	66.4	
	Mean (SD)	64.3 ( ± 12.4)	
	IQR	57.11–72.6	
**Time since Diagnosis (Years)**	Median	2.08	
	Mean (SD)	4.6 (5.2)	
	IQR	0.89–6.85	
		**n**	**%**
**Gender**	Male	486	53.7
	Female	419	46.3
**Ethnic Origin**	White	870	96.1
	Asian or Asian British	26	2.9
	Black British or Black British	7	0.8
	Unknown	2	0.02
**Disease Type**	ALL	29	3.2
	AML	67	7.4
	ANHL	54	6
	CLL	64	7.1
	CML	45	5
	HL	37	4.1
	INHL	41	4.5
	MDS	158	17.5
	MM	296	32.7
	MPN	114	12.6
**Stage of Disease**	Stable	399	44.1
	Remission	277	30.6
	Progressing	229	25.3
**Employment**	Employed	252	27.8
	Self-Employed	9	1
	Unemployed	41	4.5
	Homemaker	5	0.6
	Retired	558	61.7
	Student	10	1.1
	Other	6	0.7
	Unknown	24	2.6
**Comorbidities**	No Other Cases	533	58.9
	Other Comorbidities Cases	319	35.2
	Other Cancer	53	5.9

IQR, Inter-quartile range.

### HM-PRO Scores

The summary scores for the four domains and both scales (Part A: Impact, and Part B: signs and symptoms) are presented in **Table 2**. The mean linear score of Part A was 31.7 (95% CI = 29.6–33.8, SD = 21.6, and range 0 to 95.5) and for Part B was 20.9 (95% CI =19.9–21.8, SD = 14.2, and range 0 to 76.5). In addition, the scores for the four domains of Part A were: Physical behavior 32.1 (95% CI =30.4–33.8, SD = 26.2, and range 0 to 100); Social well-being was 24.9 (95% CI = 23.2–26.7, SD = 27.2, and range 0 to 100); Emotional behavior 38.2 (95% CI = 36.7–39.7, SD = 22.8, and range 0 to 100); and Eating and drinking 31.7 (95% CI = 29.6–33.8, SD = 31.9, and range 0 to 100). A total of 19 (2.1%) patients for Part A and 47 (5.2%) for Part B, achieved minimum score (zero) showing floor effect, however none achieved the maximum score of hundred for either part, confirming absence of ceiling effect.

**Table 2:** HM-PRO scores for the four domains, total Parts A and B.

Statistics		Physical Behavior	Social Well-being	Emotional Behavior	Eating & Drinking	Part A	Part B
Mean		32.1	24.9	38.2	31.7	31.7	20.9
95% Confidence Interval for Mean	Lower Bound	30.4	23.2	36.7	29.6	30.3	19.9
	Upper Bound	33.8	26.7	39.7	33.8	33.1	21.8
5% Trimmed Mean		30.7	22.6	37.4	29.6	30.7	20.0
Median		28.6	16.7	36.4	25.0	28.3	17.6
Variance		684.0	741.9	518.2	1,020.1	466.3	202.0
Std. Deviation		26.2	27.2	22.8	31.9	21.6	14.2
Minimum		0.0	0.0	0.0	0.0	0.0	0.0
Maximum		100.0	100.0	100.0	100.0	95.5	76.5
Interquartile Range		42.9	33.3	31.8	50.0	32.7	20.6

### Convergent Validity

The Assessment of convergent validity of both parts of the HM-PRO was performed using correlation analysis with the EORTC QLQ-C30 and FACT-G scores. Totals scores of Part A and Part B of the HM-PRO were first correlated with individual domains of EORTC QLQ-C30 and FACT-G and total Scores of FACT-G. This included correlating total score of: 1) Part A (Impact) with: five functional domains of EORTC QLQ-C30 (Physical, Role, Emotional, Cognitive, and social) and three symptom scales (Fatigue, Nausea and Vomiting, and Pain); and four domains of Fact-G (Physical, Social, Emotional, Functioning) and total Score of Fact-G; 2) Part B (Signs and Symptoms) with three symptoms scales of EORTC QLQ-C30 and three individual items (Dyspnea, Insomnia, Appetite loss); and four domains and total Score of FACT-G. The Spearman’s rank correlation coefficient estimates from this analysis are presented in **Table 3**.

**Table 3:** Correlation of total HM-PRO scores (Parts A and B) with EORTC QLQ-C30 and FACT-G domains, symptoms scale, and individual items.

Total Score of HM-PRO	EORTC QLQ C-30	Spearman’s Correlation	p Value
Part A	Physical Function	−0.71	<0.001
	Role Function	−0.72	<0.001
	Emotional Function	−0.64	<0.001
	Cognitive Function	−0.58	<0.001
	Social Function	−0.74	<0.001
	Fatigue Scale	−0.71	<0.001
	Nausea and Vomiting	−0.41	<0.001
	Pain	−0.55	<0.001
Part B	Fatigue Scale	−0.74	<0.001
	Nausea and Vomiting	−0.52	<0.001
	Pain	−0.59	<0.001
	Dyspnea	0.51	<0.001
	Insomnia	0.43	<0.001
	Appetite Loss	0.54	<0.001
** **	**FACT-G**	** **	** **
Part A	Physical Well-being	−0.74	<0.001
	Social Well-being	−0.38	<0.001
	Emotional Well-being	−0.57	<0.001
	Functional Well-being	−0.66	<0.001
	FACT-G Total score	−0.74	<0.001
Part B	Physical Well-being	−0.79	<0.001
	Social Well-being	−0.28	<0.001
	Emotional Well-being	−0.45	<0.001
	Functional Well-being	−0.57	<0.001
	FACT-G Total score	−0.65	<0.001

The total score of Part A of HM-PRO highly correlated with the five functional scales of the EORTC QLQ-C30 (Physical= −0.71, Role = −0.72, Emotional = −0.64, Cognitive = −0.58, Social = −0.74—p < 0.001). The three symptoms scales of EORTC QLQ-30 also showed moderate to high correlation (Nausea and Vomiting = −0.41, Pain = − 0.55, Fatigue = −0.71). With respect to correlation with FACT-G, the total score of Part A of the HM-PRO highly correlated with Physical (−0.74), Emotional (−0.57), Functional (−0.66) domains and overall score of FACT-G (−0.74) (**Table 3**). The correlation with Social well-being domain of FACT-G was moderate (−0.38). The negative correlation between the score of the HM-PRO and the other two instruments is observed because in EORTC QLQ-C30 and FACT-G higher scores reflect improving “Quality of life” of the patients, whereas the opposite is the case with the HM-PRO scores.

Similarly, the total score of Part B of HM-PRO highly correlated with three symptoms scales of EORTC QLQ-C30 (Fatigue scale = −0.74, Nausea and Vomiting = −0.52, Pain = −0.59—p < 0.001) and individual symptom items (Dyspnea = 0.51, Insomnia = 0.43, Appetite loss = 0.54—p < 0.001). The correlations with the four domains and total score of FACT-G were moderate to high but demonstrated weak correlation with social well-being domain (−0.28, p < 0.001) (**Table 3**). The high overall correlation between the total scores of the HM-PRO and domain/scales of EORTC QLQ-C30 and FACT-G confirmed the convergent validity of the HM-PRO.

Furthermore, the correlation between the individual domains of Part A, individual items of the HM-PRO and domains and individual items of EORTC QLQ-C30 and FACT-G was assessed. The Spearman’s rank correlation coefficient estimates from this analysis are presented in **Table 4**. The Physical behavior domain of the HM-PRO showed strong correlation of −0.79 with Physical function domain of EORTC QLQ-C30 and −0.73 with Physical well-being domain of FACT-G. The correlation of the emotional and social well-being domain of the HM-PRO with emotional and social domain of EORTC QLQ-C30 and FACT-G was also strong.

**Table 4:** Correlation of HM-PRO total scores (Parts A and B) with EORTC QLQ-C30 and FACT-G domains, symptoms scale, and individual items.

HM-PRO		EORTC QLQ C-30	Spearman’s Correlation	p Value
	**Domains**			
**Part A**	Physical Behavior	Physical Function	−0.79	<0.001
	Emotional Behavior	Emotional Function	−0.76	<0.001
	Social Well-being Eating and Drinking	Social Function Appetite	−0.55 −0.71	<0.001 <0.001
	**Individual items**			
**Part B**	Energy level	Fatigue Scale	−0.73	<0.001
	Tiredness	Fatigue Scale	−0.72	<0.001
	Back Pain	Pain Scale	−0.59	<0.001
	Body Pain	Pain Scale	−0.70	<0.001
	Breathing	Dyspnea	0.73	<0.001
	Constipation	Constipation	0.88	<0.001
	Diarrhea	Diarrhea	0.79	<0.001
	Nausea	Nausea and Vomiting Scale	−0.80	<0.001
	Sleeping	Insomnia	0.64	<0.001
	** **	**FACT-G**	** **	** **
	**Domains**			
**Part A**	Physical Behavior	Physical Well-being	−0.73	<0.001
	Emotional Behavior	Emotional Well-being	−0.75	<0.001
	Social Well-being	Social Well-being	−0.44	<0.001
** **	**Individual items**			
**Part B**	Energy level	Energy	−0.75	<0.001
	Back Pain	Pain	−0.62	<0.001
	Body Pain	Pain	−0.73	<0.001
	Nausea	Nausea	−0.82	<0.001
	Sleeping	Sleeping	−0.54	<0.001

With respect to the individual items, constipation item of the HM-PRO and EORTC QLQ-C30 showed highest correlation of 0.88. The correlations between all other similar items were above 0.5 showing strong correlation and confirming that the items of the HM-PRO measure the same construct as that of the EORTC QLQ-C30 and FACT-G items (**Table 4**).

### Univariate Regression Analysis

**Figure 1:**
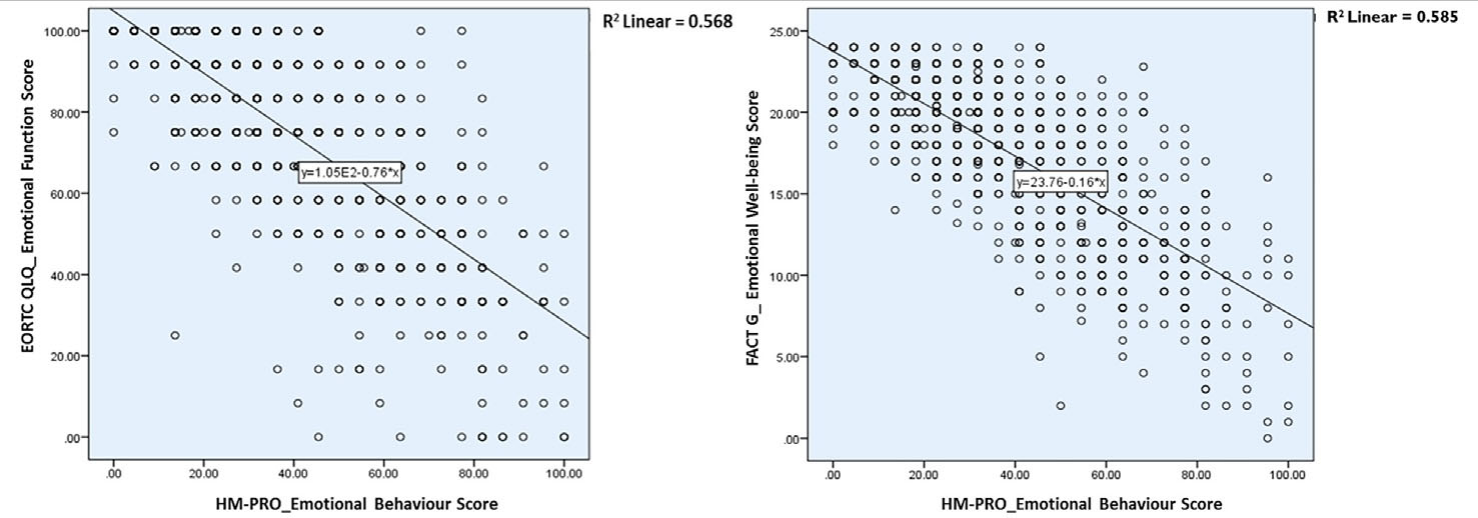
Relationship between the emotional behavior domain of the HM-PRO and emotional function scale of the EORTC and emotional well-being domain of FACT-G.

The univariate regression analysis to assess the relationship between the HM-PRO and other measure was performed in two steps: first at domain level; and second at individual item level. At the domain level the univariate regression analysis showed that the model was statistically significant indicating strong relationship between the HM-PRO domains and EORTC domains (Physical: F = 1455.13. p < 0.001, R^2^ = 62%, Emotional: F = 1,180.045, p < 0.001, R^2^ = 56%, Social: F = 360.884, p < 0.001, R^2^ = 28%, Eating and Drinking: F = 975.71, p < 0.001, R^2^ = 52%) and FACT-G (Physical: F = 937.406. p < 0.001, R^2^ = 62%, Emotional: F = 1,261.29, p < 0.001, R^2^ = 58.5%, and Social: F = 161.42, p < 0.001, R^2^ = 15.2%) (**Table 5**). The scatter plot showing the relationship between the emotional behavior domain of the HM-PRO and emotional function scale of the EORTC and emotional well-being domain of FACT-G is presented in **Figure 1**. The R^2^ value determines the strength of the relationship between the two variables i.e. “emotional well-being” domain of the HM-PRO and the respective emotional domains of the FACT-G and EORTC. Almost 60% of the variability in the FACT-G and EORTC emotional domain score is explained by the HM-PRO emotional well-being score.

**Table 5:** Univariate regression analysis of HM-PRO domains of Part A against domains of EORTC QLQ-C30 and FACT-G.

	Ind. Var (HM-PRO)	F	df	P	R2	Adj R^2^	Dep. Var	B	SE	Beta	t	p
1	**Physical Behavior**	1,455.1	1	0.0001	0.6	0.6	**EORTC_PF**	−0.7	0.02	−0.8	−38.2	0.001
							**Constant**	93.4	0.8		122.5	0.001
2	**Physical Behavior**	937.4	1	0.0001	0.5	0.5	**FACT G_PWB**	−0.2	0.01	−0.7	−30.6	0.001
							**Constant**	26.1	0.2		119.8	0.001
3	**Emotional Behavior**	1,180.1	1	0.0001	0.6	0.6	**EORTC_EF**	−0.8	0.02	−0.8	−34.4	0.001
							**Constant**	104.9	0.9		105.9	0.001
4	**Emotional Behavior**	1,261.3	1	0.0001	0.6	0.6	**FACT G_EWB**	−0.2	0.01	−0.8	−35.5	0.001
							**Constant**	23.8	0.2		117.7	0.001
5	**Social Well-being**	360.9	1	0.0001	0.3	0.3	**EORTC_SF**	−0.6	0.3	−0.5	−18.9	0.001
							**Constant**	81.6	1.2		66.8	0.001
6	**Social Well-being**	161.4	1	0.0001	0.2	0.2	**FACT-SWB**	−0.09	0.01	−0.4	−12.7	0.001
							**Constant**	22.8	0.3		88.8	0.001
7	**Eating and Drinking**	975.7	1	0.0001	0.5	0.5	**EORTC_AP**	0.6	0.02	0.72	31.2	0.001
							**Constant**	−2.3	0.9		−2.5	0.001

PB, Physical behavior; EB, Emotional behavior; SWB, Social well-being; PF, Physical Function; PWB, Physical well-being; EF, Emotional function; EWB, Emotional well-being; ED, Eating and Drinking; AP, Appetite.

Univariate regression analysis at the individual item level showed similar results with the HM-PRO items explaining more than 50% of variability when compared with all the items of the EORTC and FACT-G, except for “back pain” item of the HM-PRO with pain item of the EORTC and “sleeping” item of the HM-PRO with sleeping item of Fact-G (**Table 6**). The “constipation” item of the HM-PRO explained 74.2% of the variance of constipation item of EORTC. The models for all the compared items were statistically significant, showing strong relationship between the items of the HM-PRO and the other two instruments, confirming that they measure similar construct (**Table 6**).

**Table 6:** Univariate regression analysis of individual items of HM-PRO Part B against individual items of EORTC QLQ-C30 and FACT-G.

	Ind. Var(HM-PRO)	F	df	P	R2	Adj R2	Dep. Var	B	SE		Beta	T	P
1	**Energy Level**	1,121.9	1	0.0001	0.6	0.6	**FACTG- Energy**	−1.4	0.04		−0.8	−33.5	0.001
							**Constant**	3.6	0.04			66.9	0.001
2	**Tiredness**	980.1	1	0.0001	0.5	0.5	**EORTC_ Fatigue**	−30.9	0.1		−0.7	−31.3	0.001
							**Constant**	93.6	1.3			73.6	0.001
3	**Back Pain**	507.9	1	0.0001	0.4	0.4	**EORTC_ Pain**	−25.3	1.1		−0.6	−22.5	0.001
							**Constant**	87.7	0.9			88.2	0.001
4	**Body Pain**	985.1	1	0.0001	0.5	0.5	**EORTC_ Pain**	−31.5	1.0		−0.7	−31.4	0.001
							**Constant**	91.8	0.9			104.1	0.001
5	**Body Pain**	1,173.1	1	0.0001	0.6	0.6	**FACTG-Pain**	−1.4	0.04		−0.8	−34.3	0.001
							**Constant**	3.7	0.04			102.9	0.001
6	**Breathing**	1,075.7	1	0.0001	0.6	0.6	**EORTC_ Dyspnea**	35.8	1.1		0.7	32.8	0.001
							**Constant**	9.4	0.8			11.7	0.001
7	**Constipation**	2,575.4	1	0.0001	0.7	0.7	**EORTC_ Constipation**	36.7	0.7		0.9	50.8	0.001
							**Constant**	1.2	0.5			2.5	0.01
8	**Diarrhea**	1,701.9	1	0.0001	0.7	0.7	**EORTC_ Diarrhea**	33.9	0.8		0.8	41.3	0.001
							**Constant**	2.3	0.5			4.6	0.001
9	**Nausea**	1,145.9	1	0.0001	0.6	0.6	**EORTC_ Nausea Vomiting**	−25.1	0.7		−0.7	−33.9	0.001
							**Constant**	98.9	0.4			230.9	0.001
10	**Nausea**	1,673.5	1	0.0001	0.6	0.7	**FACTG- Nausea**	−1.3	0.03		−0.8	−40.9	0.001
							**Constant**	3.9	0.2			214.9	0.001
11	**Sleeping**	362.9	1	0.0001	0.3	0.3	**FACTG- Sleeping**	−0.9	0.04		−0.5	−19.1	0.001
							**Constant**	3.1	0.1			51.7	0.001

Ind Var, Independent variable; Dep Var, Dependent variable; F, F-Statistics; df, Degree of freedom; AdjR2, Adjusted R square value; SE, Standard error.

### Predictors of HRQoL in Hematological Malignancy

It is of importance to determine and understand the determinants of the HRQoL in patients with HMs in both research and clinical practice settings, in particular in the clinical setting where the HRQoL information might guide clinical decision-making. Multivariate regression analysis was carried out using stepwise and hierarchical regression technique to identify these predictive factors. The overall score of the Parts A and B of the HM-PRO were the dependent variable, and independent variables in the model included: Age; gender; time since diagnosis; comorbidities; occupation; ethnicity; disease diagnosed; disease state; and global question (GQ).

#### Hierarchical Regression Analysis

In the hierarchical regression analyses, two separate analysis were performed with Parts A and B as dependent variable. The independent variables were then sequentially added to the regression, one at a time, to have clear understanding on how each variable contributed to explain the variance in patient HRQoL. For Part A of the HM-PRO, the disease state explained the most variance in the HRQoL score (4.3%). Other variables making significant contribution to explaining the HRQoL included: age (2.7%); comorbidities (2.1%); and Ethnicity (0.8%). Overall the model explained 34.4% of the variance in the total score (**Table 7**). With respect to Part B, comorbidity cases explained the most variance in the HRQoL (2.2%). Other variables making significant contribution to explaining the HRQoL included: age (0.7%); and disease state (2%) (**Table 8**).

**Table 7:** Contribution of predictors explaining variance in the HM-PRO Part A scores; with hierarchical inclusion of variables.

Model	R	R Square	Adjusted R Square	SE of the Estimate	Change Statistics				
					R Square Change	F Change	df1	df2	Sig. F Change
1	0.163^a^	.03	.03	21.4	.027	23.7	1	863	.00
2	0.167^b^	.03	.03	21.4	.001	0.9	1	862	.33
3	0.171^c^	.03	.03	21.4	.001	1.3	1	861	.26
4	0.224^d^	.05	.05	21.2	.021	18.9	1	860	.00
5	0.224^e^	.05	.05	21.2	.000	0.2	1	859	.63
6	0.242^f^	.06	.05	21.1	.008	7.3	1	858	.01
7	0.242^g^	.06	.05	21.2	.000	0.04	1	857	.83
8	0.318^h^	.10	.10	20.7	.043	40.8	1	856	.00
9	0.344^i^	.12	.10	20.5	.017	16.3	1	855	.00

a Predictors: (Constant), Age.

b Predictors: (Constant), Age, Gender.

c Predictors: (Constant), Age, Gender, Time Since Diagnosis.

d Predictors: (Constant), Age, Gender, Time Since Diagnosis, Comorbidities Cases.

e Predictors: (Constant), Age, Gender, Time Since Diagnosis, Comorbidities Cases, Occupation.

f Predictors: (Constant), Age, Gender, Time Since Diagnosis, Comorbidities Cases, Occupation, Ethnicity.

g Predictors: (Constant), Age, Gender, Time Since Diagnosis, Comorbidities Cases, Occupation, Ethnicity, Disease Diagnosis.

h Predictors: (Constant), Age, Gender, Time Since Diagnosis, Comorbidities Cases, Occupation, Ethnicity, Disease Diagnosis, Stage of the Disease.

i Predictors: (Constant), Age, Gender, Time Since Diagnosis, Comorbidities Cases, Occupation, Ethnicity, Disease Diagnosis, Stage of the Disease, Global Question.

**Table 8:** Contribution of predictors explaining variance in the HM-PRO Part B scores; with hierarchical inclusion of variables.

Model	R	R Square	Adjusted R Square	Std. Error of the Estimate	Change Statistics				
					R Square Change	F Change	df1	df2	Sig. F Change
1	.081^a^	.01	.01	14.3	.007	5.7	1	863	0.02
2	.103^b^	.01	.01	14.3	.004	3.5	1	862	0.06
3	.110^c^	.01	.01	14.3	.001	1.3	1	861	0.26
4	.185^d^	.03	.03	14.1	.022	19.8	1	860	0.00
5	.187^e^	.04	.03	14.1	.001	0.4	1	859	0.49
6	.187^f^	.04	.03	14.1	.000	0.1	1	858	0.72
7	.188^g^	.04	.03	14.1	.001	0.4	1	857	0.50
8	.236^h^	.06	.05	13.9	.020	18.4	1	856	0.00
9	.257^i^	.07	.06	13.9	.010	9.2	1	855	0.00

a Predictors: (Constant), Age.

b Predictors: (Constant), Age, Gender.

c Predictors: (Constant), Age, Gender, Time Since Diagnosis.

d Predictors: (Constant), Age, Gender, Time Since Diagnosis, Comorbidities Cases.

e Predictors: (Constant), Age, Gender, Time Since Diagnosis, Comorbidities cases, Occupation.

f Predictors: (Constant), Age, Gender, Time Since Diagnosis, Comorbidities cases, Occupation, Ethnicity.

g Predictors: (Constant), Age, Gender, Time Since Diagnosis, Comorbidities Cases, Occupation, Ethnicity, Disease Diagnosis.

h Predictors: (Constant), Age, Gender, Time Since Diagnosis, Comorbidities cases, Occupation, Ethnicity, Disease Diagnosis, Stage of the Disease.

i Predictors: (Constant), Age, Gender, Time Since Diagnosis, Comorbidities Cases, Occupation, Ethnicity, Disease Diagnosis, Stage of the Disease, Global Question.

#### Backward Stepwise Regression

In the backward stepwise regression, the regression model was estimated sequentially, first all the variables were entered into the model and then the subsequent models were estimated by eliminating the least significant regressor if its significance level was ≥0.1 at each step, until there was no variable to be removed (Pope and Webster, 1972). The following predictors were retained in the final model for Part A: Disease state, ethnicity, comorbidities cases, and age. These four predictors were jointly significant in explaining Part A score of the HM-PRO by 11.5% (**Table 9**).

**Table 9:** Predictors of HM-PRO Part A score based on stepwise backward regression.

Model	R	R Square	Adjusted R Square	Std. Error of the Estimate	Change Statistics				
					R Square Change	F Change	df1	df2	Sig. F Change
1	.344^a^	.12	.11	20.5	.118	12.7	9	855	0.000
2	.344^b^	.12	.11	20.5	.000	0.01	1	855	0.95
3	.343^c^	.12	.11	20.5	.000	0.3	1	856	0.57
4	.342^d^	.12	.11	20.5	-.001	1.1	1	857	0.29
5	.340^e^	.12	.11	20.5	-.001	1.3	1	858	0.25

a Predictors: (Constant), Global Question, Stage of the Disease, Time Since Diagnosis, Ethnicity, Comorbidities Cases, Gender, Occupation, Disease Diagnosis, Age.

b Predictors: (Constant), Global Question, Stage of the Disease, Time Since Diagnosis, Ethnicity, Comorbidities Cases, Gender, Disease Diagnosis, Age.

c Predictors: (Constant), Global Question, Stage of the Disease, Time Since Diagnosis, Ethnicity, Comorbidities Cases, Gender, Age.

d Predictors: (Constant), Global Question, Stage of the Disease, Time Since Diagnosis, Ethnicity, Comorbidities Cases, Age.

e Predictors: (Constant), Global Question, Stage of the Disease, Ethnicity, Comorbidities Cases, Age.

With respect to Part B of the HM-PRO, the following predictors were retained after eliminating the variable based on backward regression: Disease state, comorbidities cases, gender, and age. These five predictors were jointly significant in explaining the variance of Part B score of the HM-PRO by 5.6% (**Table 10**). Compared to the predictors retained for Part A, Part B has gender instead of ethnicity. That means patients gender is contributing to explaining the variability in score for signs and symptoms.

**Table 10:** Predictors of HM-PRO Part B score based on stepwise backward regression.

Model	R	R Square	Adjusted R Square	Std. Error of the Estimate	Change Statistics				
					R Square Change	F Change	df1	df2	Sig. F Change
1	0.257^a^	.066	.056	13.9	.066	6.7	9	855	.000
2	0.256^b^	.066	.057	13.9	.000	0.3	1	855	.616
3	0.254^c^	.065	.057	13.9	−.001	0.8	1	856	.368
4	0.252^d^	.063	.057	13.9	−.001	1.1	1	857	.284
5	0.248^e^	.061	.056	13.9	−.002	1.8	1	858	.179

a Predictors: (Constant), Global Question, Stage of the Disease, Time Since Diagnosis, Ethnicity, Comorbidities Cases, Gender, Occupation, Disease Diagnosis, Age.

b Predictors: (Constant), Global Question, Stage of the Disease, Time Since Diagnosis, Comorbidities Cases, Gender, Occupation, Disease Diagnosis, Age.

c Predictors: (Constant), Global Question, Stage of the Disease, Time Since Diagnosis, Comorbidities Cases, Gender, Occupation, Age.

d Predictors: (Constant), Global Question, Stage of the Disease, Time Since Diagnosis, Comorbidities Cases, Gender, Age.

e Predictors: (Constant), Global Question, Stage of the Disease, Comorbidities Cases, Gender, Age.

## Discussion

The validity of an instrument explains the degree to which the instrument measures what it claims to measure and is an essential step to prove the legitimacy of the instrument. An instrument lacking validity will not answer the research question, and the outcome will be misleading. This in the context of patients with hematological malignancies might be detrimental. A non-calibrated diagnostic instrument may lead to misdiagnosis, or a non-valid HRQoL instrument may lead to under or over estimation of the impact on a patient’s functional ability, both physical and psychosocial. Thus, establishing the validity of a PRO instrument is of utmost importance. This study has provided evidence to support the validity of the HM-PRO. The majority of items, in both Parts A and B of the HM-PRO received more than 80% of affirmative responses, suggesting that the content of the instrument is relevant and important to the target patient population.

The aim of construct validity is to establish a relation with variables of other measures with which theoretically it should be associated either in a positive or a negative relation or not at all. For demonstrating the construct validity of the HM-PRO, convergent validity was examined by assessing the correlation of the similar constructs in other instrument (Streiner et al., 2015; DeVellis, 2016). The HM-PRO scores correlated with the scores of EORTC QLQ-C30 and FACT-G, at both the scale level and individual item level. The correlation coefficient had the negative value because the HM-PRO measures the impact on a patient’s HRQoL, whereas, EORTC QLQ-C30 and FACT-G measure the HRQoL.

The individual items of EORTC on constipation, diarrhea, or symptom scale like dyspnea, insomnia, and appetite loss showed positive correlation with the HM-PRO because they measure the construct in the same direction i.e. measuring the impact. The HM-PRO showed strong and significant correlation with functional scales of the EORTC and FACT-G. The individual items related to tiredness, sleeping, pain, breathing, constipation, diarrhea, nausea, and vomiting of the HM-PRO, showed strong correlation with the respective items in EORTC and FACT-G.

The univariate regression analysis conducted both at the domain and item level confirmed the strong relationship between the HM-PRO and the other two measures. For the majority of the regression models, the HM-PRO domains and individual items explained more than 50% of the variance in domain and item scores of EORTC QLQ-C30 and FACT-G, showing strong relationship and confirming the construct validity of the HM-PRO.

The HM-PRO has been developed in accordance to FDA PRO guidlines. It has shown evidence of good content validity, meaning that it captures what is important to patients with different hematological malignancies (Goswami et al., 2020). The evidence on the construct validity presented in this research is a testimony of the HM-PRO’s ability to measure HRQoL issues which it intends to measure. This is of utmost importance when a PRO is used in routine clinical practice, so that the interpretation of the score or response to an individual item is understood by the clinicians/nurses as intended by the patients. Further, the evidence supports that the HM-PRO can be used for the purpose of focusing on a specific functional area for which patient is mostly affected and might benefit from more patient-centered consultation. The clinicians have the tendency to trust their own *ad-hoc* assessment of patient HRQoL, but they are not always able to do this accurately and systematically (Basra and Shahrukh, 2009). Therefore, the HM-PRO might be useful to identify specific functional issues on individual basis early in the course of the disease and treatment.

A randomized controlled trial conducted by Basch E et al. assessed the overall survival associated with electronic patient-reported symptoms monitoring *versus* usual care (Basch et al., 2017). The authors concluded that integration of a PRO into routine care was associated with increased survival compared to usual care in patients with metastatic cancer. The potential mechanism proposed by the authors in regard to the findings is early responsiveness to patient symptoms preventing adverse consequences. The individual items of HM-PRO in Part B measuring impact of signs and symptoms have the potential to capture and monitor the responsiveness of the treatments towards these signs and symptoms across different hematological malignancies. The implementation of a PRO in routine clinical practice have shown positive outcomes in the past and with strong evidence on the content and construct validity of the HM-PRO, it has potential to focus on person-centered care and measure what is important to patients with different hematological malignancies.

## Strengths and Limitation

The HM-PRO has been developed with strong involvement of patients not only as study participants but also as a research partner and adviser. The intensive qualitative phase and robust cognitive debriefing phase ensured the content of instruments assessed for construct validity are comprehensive and covers all important aspects of HRQoL for patients with HMs. Due to the lack of a “gold standard” instrument which has been validated to be used in clinical practice for all types of HMs, the construct validation of the HM-PRO was carried out using instruments which have been developed and validated, primarily for use in clinical trials. Furthermore, most of the internationally recognized patient-reported outcome measures which are used in oncology have been developed and validated by an international patient population. Since the HM-PRO has been developed only with the UK patient population, it might not have captured certain different culture specific HRQoL issues important to such patients. Although, certain aspects of translatability and universality were taken into account during relevant stages of development, such issues should be considered while translating and cross-culturally adapting the HM-PRO into different languages/cultures.

## Data Availability Statement

The raw data supporting the conclusions of this article will be made available by the authors, without undue reservation.

## Ethics Statement

The studies involving human participants were reviewed and approved by NRES South West Bristol, UK (ref 14/SW/0033). The patients/participants provided their written informed consent to participate in this study.

## Author Contributions

PG collected the data, developed the analysis policy, liaised with hospitals for patient recruitment, analyzed the data, interpreted results, and wrote the first draft of the manuscript. RE contributed to data collection as a patient research partner and reviewed the draft manuscript. SS generated the original idea, developed the study protocol, supervised the study, liaised with study centers as part of patient recruitment, developed the analysis policy, interpreted results, and reviewed the draft manuscript. EO and TI contributed to the design of the study, interpreted results, and reviewed the draft manuscript. JK, AF, DJ, MK, SA-I, MA-O, MA, GC, SM, CL, MA, and MO contributed to patient recruitment from their respective center and reviewed the draft manuscript. All authors contributed to the article and approved the submitted version.

## Funding

The study was funded by the European Hematology Association Scientific Working Group “Quality of life and Symptoms” through unrestricted grants from Novartis, Bristol Myers Squib, and Sanofi. Funders had no role in study design, data collection and analysis, decision to publish, or preparation of the manuscript.

## Conflict of Interest

The authors declare that the research was conducted in the absence of any commercial or financial relationships that could be construed as a potential conflict of interest.

## Abbreviations

ALL, Acute Lymphoid Leukemia; AML, Acute Myeloid Leukemia; ANHL, Aggressive Non-Hodgkin Lymphoma; CI, Confidence Interval; CLL, Chronic Lymphoid Leukemia; CML, Chronic Myeloid Leukemia; HL, Hodgkin Lymphoma; HM, Hematological Malignancy; HRQoL, Health-Related Quality of life; INHL, Indolent Non-Hodgkin Lymphoma; MDS, Myelodysplastic Syndromes; MM, Multiple Myeloma; MPN, Myeloproliferative Neoplasm; n, Number of Patients Recruited; QoL, Quality of Life; SD, Standard Deviation.
